# No statistical learning advantage in children over adults: Evidence from behaviour and neural entrainment

**DOI:** 10.1016/j.dcn.2022.101154

**Published:** 2022-09-21

**Authors:** Christine N. Moreau, Marc F. Joanisse, Jerrica Mulgrew, Laura J. Batterink

**Affiliations:** Western University, Brain and Mind Institute, Perth Dr, London, ON N6G 2V4, Canada

**Keywords:** Statistical learning, Language acquisition, Neural entrainment, Developmental differences, Implicit learning, Explicit learning

## Abstract

Explicit recognition measures of statistical learning (SL) suggest that children and adults have similar linguistic SL abilities. However, explicit tasks recruit additional cognitive processes that are not directly relevant for SL and may thus underestimate children’s true SL capacities. In contrast, implicit tasks and neural measures of SL should be less influenced by explicit, higher-level cognitive abilities and thus may be better suited to capturing developmental differences in SL. Here, we assessed SL to six minutes of an artificial language in English-speaking children (*n* = 56, 24 females, *M* = 9.98 years) and adults (*n* = 44; 31 females, *M* = 22.97 years), using explicit and implicit behavioural measures and an EEG measure of neural entrainment. With few exceptions, children and adults showed largely similar performance on the behavioural explicit and implicit tasks, replicating prior work. Children and adults also demonstrated robust neural entrainment to both words and syllables, with a similar time course of word-level entrainment, reflecting learning of the hidden word structure. These results demonstrate that children and adults have similar linguistic SL abilities, even when learning is assessed through implicit performance-based and neural measures.

## Highlights

• Developmental differences in statistical learning were examined.
• Both children and adults showed behavioural evidence of word learning.
• Children and adults showed robust neural entrainment to words and syllables.
• Children and adults showed an increase in neural entrainment to words over time.

Language learning is a challenging task given that continuous streams of sounds must be segmented into meaningful units of speech. Seemingly effortlessly, infants acquire spoken language simply by listening to others speak, with little recourse to explicit learning strategies. It is thought that *statistical learning (SL)* – the ability to pick up on regular or quasi-regular patterns in the environment through passive exposure to input – plays an important role in language acquisition (Kuhl, 2004, Seidenberg, 1997, Saffran et al., 1996b). Indeed, early studies of SL showed that 8-month-old infants and adults can segment words in a continuously presented artificial language, presumably by rapidly gaining sensitivity to the transitional probabilities between neighbouring syllables (e.g., Saffran et al., 1996a).

## 1 The developmental trajectory of statistical learning

While SL is especially noted for its proposed role in language learning (Bates and Elman, 1996, Romberg and Saffran, 2010), it is a domain-general phenomenon (Aslin, 2017), observed in learning linguistic and nonlinguistic stimuli including syllables (Saffran et al., 1996a), auditory tones (Saffran et al., 1999), visual shapes (Fiser and Aslin, 2002), and tactile patterns (Conway and Christiansen, 2005). SL is typically quantified by first exposing participants to a continuous stream of repeating patterns (such as trisyllabic nonsense words or shape triplets) and then asking them to discriminate between items from the stream and recombined foil items using a two-alternative forced choice (2AFC) recognition task. When this type of measure is used, both visual and non-linguistic auditory SL have been found to improve over childhood, from ages 5–12 (Arciuli and Simpson, 2011, Shufaniya and Arnon, 2018; Raviv and Arnon, 2018) and from ages 6–30 (visual SL; Schlichting et al., 2017).

However, growing evidence suggests that the developmental trajectory of SL may differ for linguistic stimuli, with SL of syllable patterns showing *stable* rather than improving 2AFC performance across ages (McNealy et al., 2010, McNealy et al., 2011, Raviv and Arnon, 2018, Saffran et al., 1997). In particular, Raviv and Arnon (2018) directly compared the developmental trajectories of visual and linguistic SL from ages 5–12 and found that children’s learning in the visual domain improves with age, whereas learning linguistic stimuli did not change across ages. Similarly, no performance differences were found between 6 and 7-year-olds and adults on a linguistic SL task (Saffran et al., 1997). However, an important caveat is that in Raviv and Arnon’s (2018) study, the youngest age group (i.e., 5- to 6-year-olds) did not exceed chance and in the McNealy et al., 2010, McNealy et al., 2011 studies, *none* of the age groups performed above chance. These results suggest that the 2AFC task, which requires explicit decision-making, may be poorly suited to capturing SL in younger children. In addition, given that older children outperform younger children on 2AFC tests of non-linguistic stimuli, one theoretical possibility is that younger children may have a true *linguistic* SL advantage, which is obscured by the task demands of the 2AFC test.

This possibility is supported by previous findings showing that adult learners’ knowledge of their native language influences – and often impedes – SL of a novel artificial language, reflecting “linguistic entrenchment” (Finn and Hudson Kam, 2008, Siegelman et al., 2018, Trecca et al., 2019). Relative to adults, children have less experience with their native language, and thus may be more easily able to flexibly adapt to new representations that potentially conflict with prior linguistic knowledge (Thiessen et al., 2016). In addition, success in acquiring many other key aspects of language, such as phonetic contrasts and complex morphology and syntax, declines as a function of age, with younger children achieving native like fluency at much higher rates than older learners (Hakuta et al., 2003, Hartshorne et al., 2018, Johnson and Newport, 1989, Werker and Tees, 1984). Given these findings, it may be the case that linguistic SL follows a different developmental trajectory than other forms of SL.

In the present study, we evaluate the possibility that children have an underlying linguistic SL advantage that is obscured by the 2AFC task typically used to assess learning. Previous work suggests that the 2AFC task is not sufficiently reliable in children (Arnon, 2019) to reveal a relationship between SL and age. Further, this task primarily measures explicit knowledge, and may therefore underestimate implicit learning (Batterink et al., 2015). This may disproportionately disadvantage children, who arguably benefit more from implicit rather than explicit learning (Finn et al., 2015). Recent work has also shown that implicit measures are more reliable at the individual level compared to the 2AFC task (Kidd et al., 2020). Therefore, a combination of more sensitive implicit and behaviourally independent measures may provide better insight into SL in younger ages.

### 1.1 Capturing linguistic statistical learning with EEG-based neural entrainment

One possible avenue of progress is to capture linguistic SL with EEG or MEG-based *neural entrainment* (Buiatti et al., 2009), the phase-locking of neural oscillations to rhythmic or quasi-rhythmic stimuli. In the context of SL, neural entrainment can be used to capture the implicit learning of linguistic regularities by presenting artificial language stimuli at a steady, isochronous rate. As the embedded words in the speech stream are learned, increasing entrainment at the word presentation frequency should be observed (Buiatti et al., 2009). Because this measure does not require a behavioural response, it may provide a robust way to index and compare SL across different age groups.

Recent work supports this approach. Previous SL studies (Batterink and Paller, 2017, Batterink and Paller, 2019, Batterink and Choi, 2021) observed word-level neural entrainment in adults using inter-trial phase coherence (ITC), a measure of event-related phase locking (Tallon-Baudry et al., 1996). Word-frequency ITC was found to increase over the course of exposure to the speech stream, while syllable-frequency ITC was found to decrease over the same time-period. These changes in entrainment are thought to reflect a perceptual shift from processing individual raw syllables in favour of coherent words and can be quantified using the *Word Learning Index (WLI)*, computed as the ratio of word-level to syllable-level ITC. Individuals’ WLI correlated with their performance on an implicit reaction-time based measure of SL, supporting its validity as an index of statistical word knowledge (Batterink and Paller, 2017, Batterink and Paller, 2019).

Of close relevance to the current study, Choi et al. (2020) used this neural entrainment approach to directly compare the time course of SL in 6-month-old infants and adults. Their findings indicated that infants and adults exhibit a similar increase in the WLI over the exposure period, suggesting comparable linguistic SL abilities between these two ages groups. Here, infants’ WLI also predicted their subsequent looking time behaviour to novel words. However, while these findings provide initial evidence that linguistic SL is stable between infancy and adulthood, our understanding of the full developmental trajectory of SL remains far from complete. There are profound changes that occur throughout childhood in cognitive processes such as attention (Lin et al., 1999), working memory (Gathercole et al., 2004), and long-term memory (Unsworth, 2019), all of which have been implicated as underlying components in SL (Arciuli, 2017, Conway, 2020). Given these changes, one possibility is that SL abilities follow an inverted-U trajectory, in which childhood represents a “sweet spot” for SL relative to earlier and later periods (Gualtieri and Finn, 2022). In addition, infants’ ability to perform behavioural tasks is limited, making it difficult to comprehensively assess both explicit and implicit knowledge in this age group.

### 1.2 Current study

Motivated by these issues, the current study aimed to comprehensively characterise and compare SL during middle childhood (ages 8–12) and adulthood (17–49 years), using an EEG-based measure of neural entrainment during exposure, as well as both explicit and implicit measures of learning post-exposure. Incorporating multiple measures of learning, rather than using only a single explicit task such as the 2AFC recognition task, may produce a clearer understanding of SL across development.

## 2 Methods

### 2.1 Participants

Participants were recruited via a participant pool, posters, and social media posts in London, Ontario. Informed consent was obtained from all adult participants, and children’s parents. Assent was obtained from child participants.

#### 2.1.1 Children

A total of 56 native English speakers ages 8–12 participated (24 females, *M* = 9.98 years, *SD* = 1.26 years, range: 8;1–12;8 years). This age range was selected to ensure that most children would be able to successfully complete the implicit target detection task. One child’s data were excluded from the EEG analyses due to a technical error with data recording.

#### 2.1.2 Adults

We relied on a convenience sample of existing data for the adult analyses. Two partially overlapping samples of adult English speakers were used for behavioural (*n* = 40; 31 females; *M* = 22.77 years, *SD* = 7.14 years, range: 17–49 years; sample “A” & “B” below) and EEG (*n* = 24; 14 females; *M* = 19.75 years, *SD* = 3.26 years, range: 17–32 years; sample “B” & “C”) age-group comparisons. We initially recruited 20 adult participants to complete the same behavioural protocol as the children (sample A). An additional 20 participants were added from a recently collected sample not previously reported (sample B; Mulgrew & Batterink, in prep). Sample B participants completed the EEG protocol and the rating and target detection tasks but did not complete the 2AFC task. An additional 4 adult participants drawn from a previously published study (sample C; Batterink and Paller, 2017) were added to the EEG sample to increase power for EEG analyses. Due to counterbalancing in the previous 2017 study, only these 4 particular participants were selected as they had been exposed to a very similar language used in the current study (detailed in Section 2.2.1). No behavioural data were available for sample C. All adult participants were exposed to a highly similar or identical language as the children.

### 2.2 Tasks

**Fig. 1:**
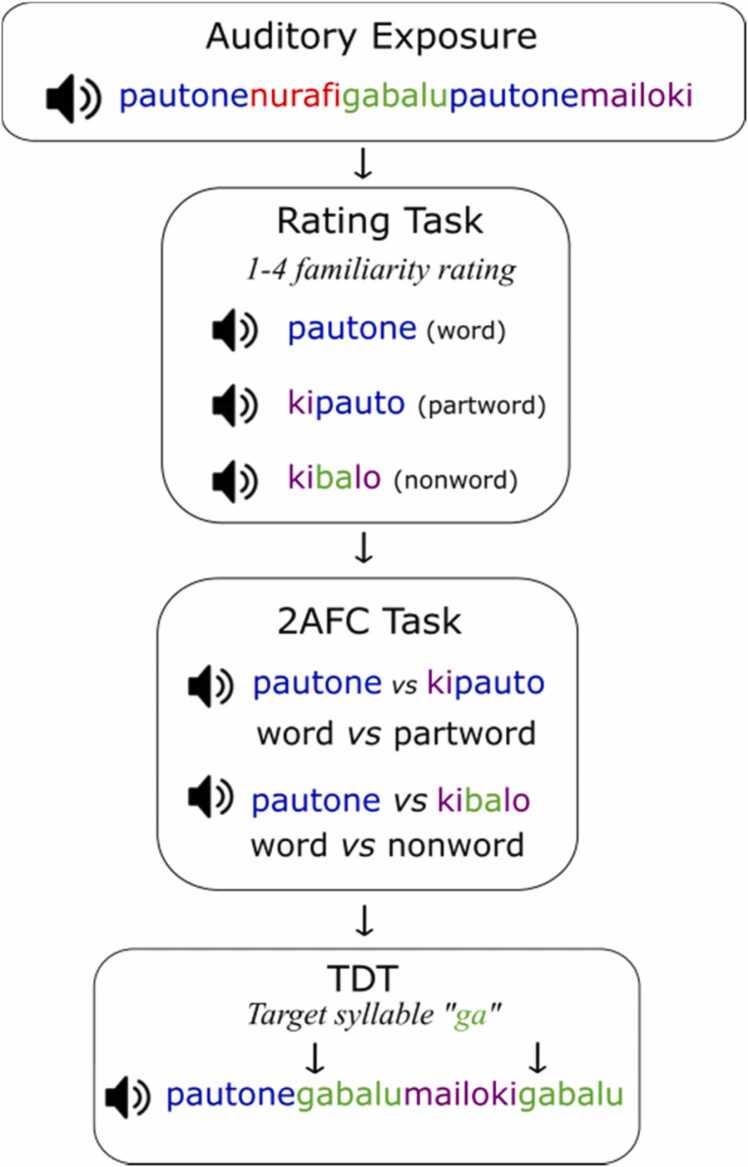
Behavioural protocol. Auditory SL tasks were completed in the order shown. (Note that Adult Sample B did not complete the 2AFC task).

All participants were passively exposed to an auditory speech stream. After the exposure task, all children (*n* = 56), as well as adults in the behavioural group (*n* = 40), completed behavioural tasks designed to assess implicit and explicit knowledge of the artificial language (see Fig. 1 for summary).

#### 2.2.1 Artificial language exposure

Stimuli consisted of 12 synthetic speech syllables modified from Batterink and Paller (2017), recorded at a sampling rate of 44100 Hz. Syllables were combined into an artificial language consisting of four unique trisyllabic words (children and adult sample A: *pautone, nurafi, gabalu,* and *mailoki;* adult sample B*: pautoki, nurafi, gabalu,* and *mailone;* adult sample C: *pautone, nurafi, gamilu,* and *maipuki*). The four words were concatenated into a six-minute continuous speech stream, such that each word immediately followed the next, with no acoustic word onset cues. Each of the four words was presented 100 times at a rate of 300 ms per syllable, for a total of 400 words. Presentation order was pseudorandom, with the only restriction that the same word could not repeat twice in a row. As such, the transitional probability of neighbouring syllables within words was higher than between words (within: 1.00; between: 0.33).

While passively listening to the auditory stream, children watched a silent six-minute video clip of “Shaun the Sheep” to prevent boredom (Cary and Symanowski, 2008). The video was not aligned to the auditory stimuli, such that across children, any eye movements or neural responses to the video would not systematically influence electrophysiological responses to the speech stream. Adults in the behavioural group coloured in an adult colouring book, while adults in the EEG group viewed a static image on a computer monitor.

#### 2.2.2 Rating task

This task requires participants to make a graded familiarity judgement for each item, providing a measure of explicit knowledge. Following the procedure used by Batterink and Paller (2017), participants heard a three-syllable word or foil and provided a familiarity rating on a 1–4 scale (1- “very unfamiliar”, 4 - “very familiar”). A total of 12 trials were presented, consisting of the four words from the speech stream, four partword foils and four nonword foils. Partwords were created by adding a syllable to a syllable pair (i.e., adding the final syllable of one word to the first two syllables of another word or adding the first syllable of one word to the final two syllables of another word). Nonwords were made up of syllables that never occurred together in the speech stream. The exact partword and nonword foils of adult sample B differed from those used in children and adult sample A, which we considered as a factor in later analyses. A repeated measures ANOVA was used to examine the effect of word category (word, partword and nonword) on familiarity ratings, with age group (children and adults) and test version (children & sample A, sample B) as between-subjects factors. To compute correlations with other variables, a composite “rating score” was calculated by subtracting the mean score for partwords and nonwords from the mean score for words. Data from one child were excluded because he reported afterwards that he did not understand the task and pressed the same key for each trial.

#### 2.2.3 Two-alternative forced choice (2AFC) recognition task

This task requires participants to explicitly discriminate between words from the stream and foil items, providing an additional measure of explicit knowledge. Following the procedure used by Batterink et al. (2015), the participants heard a word from the training set and a nonword or partword foil separated by a 1500 ms pause. They then indicated which word was most familiar (1 or 2) and provided a remember/familiar/guess response (results for R/F/G responses reported in Supplementary materials; see Fig. S1). The words were exhaustively paired with two partwords and two nonwords for a total of 16 trials. For adults, only sample A completed this task. A one-sample t-test was conducted to determine whether performance exceeded chance. A one-way ANOVA examined whether there were significant differences between children and adults.

#### 2.2.4 Target detection task

This task requires participants to make speeded responses to target syllables presented with structured speech streams, providing a reaction time (RT)-based measure of implicit statistical knowledge (Batterink et al., 2015). Each speech stream contained the four words from the language repeated four times each in pseudorandom order, with no item presented twice in immediate succession. Prior to each stream, participants heard the target syllable in isolation and were instructed to respond with a button press every time they heard the target syllable.

The adult version of the task included 36 speech streams, in which words were presented at the same rate as the original exposure stream (300 ms/syllable) for a total of 48 targets in each syllable position. To reduce overall length of the task, the child version of the task included 24 speech streams for a total of 32 targets in each syllable position. The syllable presentation rate was slowed by 50 ms (350 ms/syllable) to ensure that children were able to successfully complete the task. To keep children’s attention on the task and to decrease haphazard responding, a square at the bottom of the screen changed colour with each button press. The children and adult sample B also completed three practice trials to ensure understanding of the task, in which they heard syllables that were not part of the artificial language. Only responses 0–1400 ms of syllable onset for the children and 0–1200 ms for the adults (corresponding to the duration of 4 syllables in each case) were considered accurate and included in RT analyses. A repeated measures ANOVA was used to test the effect of syllable position (1st, 2nd, 3rd) on RTs, with age group (children and adults) included as a between-subjects factor.

Mean RTs for correct responses were calculated for each syllable position (initial, middle, and final). If learners have become sensitive to the statistical structure of the speech stream, word-final syllables should elicit the fastest RTs, as these syllables can be implicitly predicted based on the preceding syllables (Batterink et al., 2015). The RT priming effect was computed as the difference in RTs between the final and first syllables, divided by the RT for the first syllable [(S_1_ – S_3_)/S_1_] (Batterink and Paller, 2019). This calculation adjusts for potential differences in baseline RTs, allowing us to compare implicit SL across participants with different RT baselines. An independent-samples t-test was used to examine whether the RT priming effect differed between age groups. The hit rate was also calculated (number of targets hit divided by number of total targets), as well as the normalised number of false alarms (total number of false alarms/number of actual targets). This equated for the different length of the task between children and adults. One child did not complete this task.

### 2.3 EEG recording procedures

EEG was recorded during the exposure of the novel language using a Biosemi Active-Two system. For the children, 32 Ag/AgCl-tipped electrodes attached to an electrode cap, placed according to the International 10–20 system, were used to record EEG. Two electrodes were placed on the left and right mastoid. Six electrooculogram (EOG) electrodes were placed under, above and next to each of the eyes. Due to using a convenience sample of adult participants, there are moderate differences in recording montage. For adults, there were 64 instead of 32 scalp electrodes and two instead of six EOG electrodes, placed under and next to the left eye.

Data were processed using EEGLAB (Delorme and Makeig, 2004) and the ERPLAB open-source toolbox (Lopez-Calderon, and Luck, 2014). EEG signals were recorded relative to the Common Mode Sense (CMS) active electrode and re-referenced offline to the average of the left and right mastoids. EEG was recorded at a sampling rate of 512 Hz and was filtered offline using a 60 Hz notch filter and a band-pass filter from 0.1 to 20 Hz. The continuous EEG was manually inspected, and artifacts judged to be extracranial in origin were rejected. Independent components analysis was then used to detect and remove eye blinks and eye movement artifacts. Adults in sample C were exposed to a total of 12 min (800 words) of the artificial language, but only EEG data from the first 6 min (400 words) were extracted for the current analyses, equating total exposure duration for all participants.

To analyze neural entrainment across the exposure period, data were time-locked to the onset of each word and extracted into nonoverlapping epochs each containing 12 words (10.8 s). The Fast Fourier Transform (FFT) was then applied to each epoch, and inter-trial phase coherence (ITC) was computed across all epochs for each frequency bin of interest, ranging from 0.6 to 5 Hz, with a bin width of 0.09 Hz (following the analysis outlined by Benjamin et al., 2021; Batterink and Choi, 2021). ITCs ranged from 0 to 1, with 0 indicating purely non-phase-locked activity and 1 indicating strictly phase-locked activity (i.e., oscillations perfectly in phase across all epochs).

**Fig. 2:**
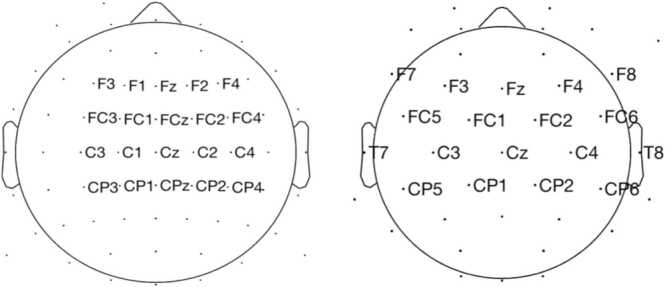
Layout of electrodes used in the computation of the ITC and WLI values in children (right) and adults (left).

For auditory tasks, neural entrainment and learning effects are broadly maximal over frontocentral electrodes, with the smallest effects occurring over occipital regions. To avoid diluting our effects of interest, we selected frontocentral electrodes where EEG signal were generally maximal for each group, covering a similar yet somewhat broader area in children (see Fig. 2 for exact electrodes; children = 18 out of 32 electrodes; adults = 20 out of 64 electrodes).

We were specifically interested in ITC at two frequencies of interest: the word frequency (ITC_word_; 1.1 Hz) and the syllable frequency (ITC_syllable_; 3.3 Hz). These values were also used to compute a composite neural measure of SL, the Word Learning Index (WLI = ITC_word_ divided by ITC_syllable_; Batterink and Paller, 2017, Batterink and Paller, 2019; Choi et al., 2020, Chen et al., 2020). Higher WLI values indicate greater neural entrainment towards the triplet frequency relative to the raw syllable frequency, reflecting stronger sensitivity to the words embedded in the artificial language and better SL.

#### 2.3.1 Overall neural entrainment effects across exposure

To establish statistical significance of ITC peaks, we carried out a surrogate analysis that established which effects might be expected under the null hypothesis of non-entrained oscillatory activity. For each participant, a single control surrogate dataset was derived from their actual dataset by jittering each word onset relative to the actual original word onset by a randomly selected integer value ranging between − 900–900 ms. This procedure removed the consistent relationship between the EEG signal and the auditory speech stream while preserving each epoch’s general timing within the exposure period. As with the original datasets, epochs were extracted at surrogate word onset, and ITCs were computed to produce an estimate of ITC across frequencies under a null model of non-entrainment. Paired-samples t-tests were then used to determine whether actual ITC values significantly exceeded the surrogate ITC values across participants, within each group.

Finally, Pearson’s correlations were conducted between overall neural entrainment (WLI, ITC_word_ and ITC_syllable_) and our three behavioural measures (rating score, RT priming effect, and 2AFC recognition accuracy) to determine whether neural entrainment during exposure predicts subsequent SL performance (see Supplementary Material; Fig. S4, Table S5, Table S6).

#### 2.3.2 Time course analysis

For the time course analysis, we used a sliding-time-window to examine the trajectory of learning across the 6 min of exposure. To estimate learning across time, we created “bundles” containing 5 nonoverlapping epochs, with each bundle shifted by one epoch (e.g., epochs 1–5, 2–6, 3–7, etc.), such that each bundle represents 54 s of learning. ITC within each bundle was then calculated using the same method as described previously for the overall ITC analysis. ITC values were then averaged across our channels of interest (Fig. 2) to produce an estimate of phase-locking at each frequency within each bundle. Within each bundle, ITC at the word and syllable frequencies were used to compute the WLI, as in the original analysis. ITC values at the word and syllable frequencies and the WLI within each bundle were plotted, reflecting the progression of learning across the exposure period. Linear mixed-effects modelling was used to test the hypothesis that the WLI will increase as a function of exposure, reflecting the progression of learning over time. The WLI was computed for each bundle and each participant and classified according to participant and bundle number (i.e., 1–28). Several bundles near the end of the exposure period did not contain data from all participants; bundles missing data from more than 50 % of participants were excluded from the analyses, leaving bundles 1–26 for final analysis. Within each group (children, adults), the WLI was modelled separately, with bundle number included as a fixed effect and participant included as a random intercept.

In addition, to directly compare the time course of neural entrainment between age groups, a model was tested that included the WLI as the dependent variable, bundle, group, and the interaction between bundle and group as fixed effects, and participant as a random intercept. A significant interaction between age group and bundle would provide evidence that the temporal progression of learning, or gain in sensitivity to the embedded words, varies as a function of age. To further evaluate the effect of age on the time course of learning, within the children’s group, we conducted an additional linear mixed-effects model (see Supplementary Material, Table S4), with age, bundle, and the interaction between age and bundle as fixed effects and participant included as the random intercept.

To fully characterise any potential differences in neural entrainment between groups, the same models as described above were also conducted with ITC_word_ and ITC_syllable_ as the dependent variables. Our prediction was that ITC_word_ should increase and ITC_syllable_ should decrease as a function of exposure, which would logically lead to an increase in the WLI.

## 3 Behavioural results

### 3.1 Rating task

**Fig. 3:**
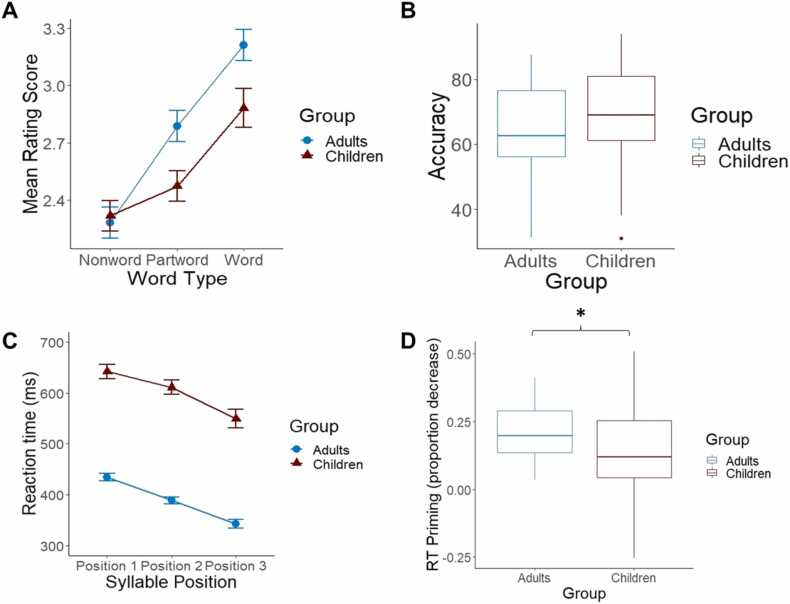
Behavioural performance in children and adults. (A) Performance on the familiarity rating task. Mean rating (out of 4) for word type (word, partword, nonword). Error bars represent standard error. (B) Boxplots of the performance on the 2AFC recognition task. (C) Performance on the target detection task. Mean RTs for each syllable position. Error bars represent standard error. (D) Boxplots of the RT priming effect (S_1_-S_3_/S_1_) on the target detection task. * Indicates a significant difference (p < .05) in RT priming effect between children and adults.

Both children and adults rated words as most familiar, followed by partwords, with nonwords rated as least familiar (Word Category Effect: Children: *F*(2, 108) = 28.05, *p* < .001, *η*_*p*_^*2*^ = .342; linear contrast: *F*(1,54) = 44.09, *p* < .001, *η*_*p*_^*2*^ = .449; Adults: *F*(2, 78) = 48.49, *p* < .001, *η*_*p*_^*2*^ = .554; linear contrast: *F*(1,39) = 76.23, *p* < .001, *η*_*p*_^*2*^ = .662; see Fig. 3 A). Performance on the rating task was not significantly different between test versions in the adult group (Adult Test Version x Word Category Effect: *F*(2, 76) = 1.16, *p* = .318, *η*_*p*_^*2*^ = .030). Although the word category effect appeared numerically larger in adults, after controlling for test version, adults and children did not significantly differ in performance (Group x Word Category Effect: *F*(2184) = 1.79, *p* = .170, *η*_*p*_^*2*^ = .019). Similar results were found when comparing children to adult sample A alone (who were tested on identical experimental stimuli); see Supplementary materials.

### 3.2 2AFC recognition task

Both children and adults performed significantly above chance (50%) on the recognition task (see Fig. 3B; Children: *M* = 67.91 %, *SD* = 15.34 %, *t*(55) = 33.12, *p* < .001, Cohen’s d = 4.426; Adults: *M* = 65.63 %, *SD* = 13.53 %, *t*(19) = 5.17, *p* < .001, Cohen’s d = 1.155). Recognition performance did not significantly differ between groups, *F*(1, 74) = 0.347, p = .558, *η*_*p*_^*2*^ = .005.

### 3.3 Target detection task

Both children and adults showed progressively shorter RTs for the first, second and third syllables, reflecting facilitation due to SL (see Fig. 3C; Syllable Position Effect: Children: *F*(2108) = 19.54, *p* < .001, *η*_*p*_^*2*^ = .266; linear contrast: *F*(1,54) = 36.39, *p* < .001, *η*_*p*_^*2*^ = .403; Adults: *F*(2, 78) = 100.86, *p* < .001, *η*_*p*_^*2*^ = .721; linear contrast: *F*(1,39) = 163.42, *p* < .001, *η*_*p*_^*2*^ = .989). The syllable position effect did not differ between groups (Group x Syllable Position: *F*(2186) = 0.44, *p* = .644, *η*_*p*_^*2*^ = .005; linear contrast: *F*(1,93) = 0.002, *p* = .964, *η*_*p*_^*2*^ = .000), although adults responded significantly faster than children overall (Main Effect of Group: *F*(1,93) = 172.19, *p* < .001, *η*_*p*_^*2*^ = .725). Similar results were found when comparing children to adult sample A alone (who were tested on identical experimental stimuli); see supplementary materials.

Adults showed a significantly larger RT priming effect than children, indicating that when baseline RTs are considered, the *relative* speed-up to more predictable syllables is larger in adults than children (*t*(93) = 2.34, *p* = .021, Cohen's d = 0.487; see Fig. 3D).

Children’s average hit rate was 71.3 % (*SD* = 13.0%) and adults’ average hit rate was 88.5 % (*SD* = 6.5 %). Children’s hit rate was significantly lower than adults’ (*F*(1,93) = 57.45, *p* < .001, *η*_*p*_^*2*^ = .382). Further, the normalised false alarm rate was significantly greater in children (*M* = 0.23, *SD* = 0.14) than in adults (*M* = 0.09, *SD* = 0.09; *F*(1, 93) = 29.21, *p* < .001, *η*_*p*_^*2*^ = .240). In sum, adults detected syllables more quickly and accurately than children overall, and had a greater RT priming effect, indicative of a greater proportional facilitation effect.

## 4 EEG results

### 4.1 Overall neural entrainment effects across exposure

**Fig. 4:**
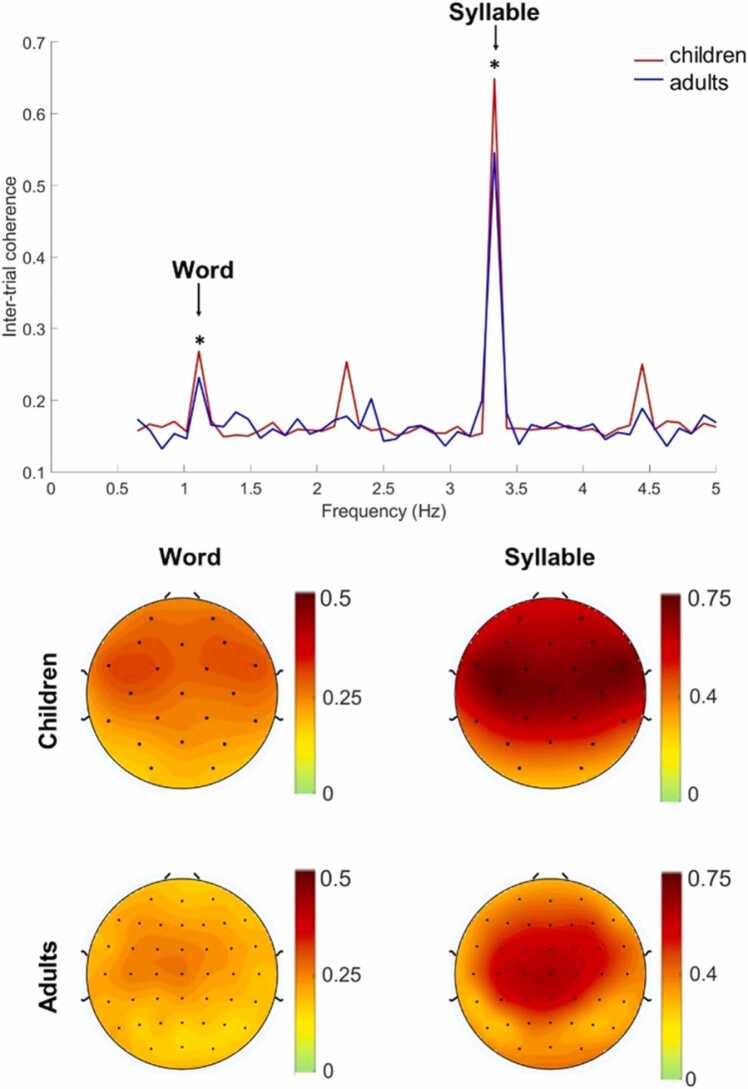
Neural entrainment at frontocentral electrodes for the children and the adults over the six-minute exposure period. ITC as a function of frequency reveal clear peaks at frequencies corresponding to the word rate (1.1 Hz) and syllable rate (3.3 Hz). *Indicates significant ITC compared to surrogate values. The bottom image demonstrates topographical plots of the distribution of ITC at the word and syllable frequencies across the scalp.

As expected, both children and adults showed clear peaks in ITC at the word and syllable frequencies (Fig. 4; see also Fig. S1 and Table S1 for ITC data relative to null-estimate surrogate data). In both groups, observed ITC values significantly exceeded surrogate ITC values, at both word and syllable frequencies, indicating significant neural entrainment above baseline (Children: Word: *t*(54) = 7.49, *p* < .001, Cohen’s d = 1.010; Syllable: *t*(54) = 23.19, *p* < .001, Cohen’s d = 3.127; Adults: Word: (*t*(23) = 3.72, *p* = .001, Cohen’s d = 0.760; Syllable: *t*(23) = 11.42, *p* < .001, Cohen’s d = 2.330).

Because ITC values may be influenced by age-related anatomical differences such as skull thickness and cortical volume, any significant age group differences in overall ITC values are not straightforward to interpret. Following the previous approach comparing neural entrainment in infants and adults (Choi et al., 2020), we restricted our statistical comparisons between age groups to the learning trajectories over time, as revealed by changes in neural entrainment across the exposure period.

### 4.2 Time course of neural entrainment

**Fig. 5:**
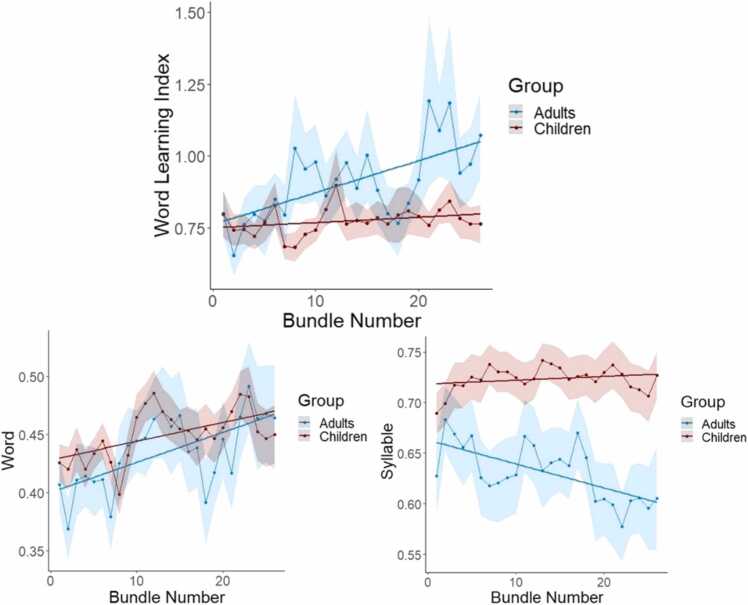
Time course of neural entrainment for children and adults according to bundle number. The top graph shows our primary neural entrainment measure, the word learning index (WLI), computed as the ratio of word entrainment relative to syllable entrainment, as a function of exposure. The bottom graphs show ITC at the word frequency (left) and at the syllable frequency (right) as a function of exposure.

Table 1 reports the time course of neural entrainment results in children and adults, including the parameter estimate indicating change in ITC across each bundle. In adults, as expected, the WLI significantly increased over the course of exposure. Further analyses indicated that the WLI increase corresponded to both a significant increase in ITC_word_ and a significant decrease in ITC_syllable_ over the course of exposure. Children also showed a significant increase in ITC_word_; however, there was no significant decrease in ITC_syllable_ and only a marginally significant increase in the WLI (see Fig. 5).

**Table 1:** Time course of neural entrainment in children and adults. The table reports the linear mixed-effects model results for the WLI, ITC_word_, ITC_syllable_ reported by group (children and adults). Parameter estimate indicates the increase or decrease as a function of bundle.

Group	ITC	*df*	*F*	*p*	Parameter estimate (SE)
Children	WLI	1,1240	3.04	0.081	2.73 × 10^−3^ (1.57 × 10^−3^)
	Word	1, 1246	12.49	> 0.001	1.79 × 10^−3^ (5.06 × 10^−4^)
	Syllable	1,1237	0.258	0.612	1.88 × 10^−4^ (3.71 × 10^−4^)
Adults	WLI	1554	8.20	0.004	0.01 (3.55 × 10^−3^)
	Word	1554	5.96	0.015	2.14 × 10^−3^ (8.76 × 10^−4^)
	Syllable	1551	10.88	0.001	-2.57 × 10^−3^ (7.79 × 10^−4^)

Children and adults showed a similar time course of entrainment to the word structure, as indexed by the progression of ITC_word_ across bundles (Table 2; see Fig. 5). However, there was a significant group difference in the time course of entrainment at the syllable level, with adults demonstrating a greater decrease in ITC_syllable_ over time compared to children (Table 2). There was also a significant difference in the progression of the WLI between children and adults (Table 2), which may be accounted for by the difference in syllable level entrainment.

**Table 2:** Linear mixed-effects model results for adults compared to children for the WLI, ITC_word_, ITC_syllable_ for the group x bundle interaction and main effects of group and bundle. Positive parameter estimates for the interaction indicate greater entrainment over time for adults relative to children.

ITC	Effect	df	F	p	Parameter estimate (SE)
WLI	Group x Bundle	1, 1795	4.91	0.027	7.39 × 10^−3^ (3.35 × 10^−3^)
	Group	1, 143	0.115	0.735	0.02 (0.08)
	Bundle	1,1795	14.76	< 0.001	2.71 × 10^−3^ (1.85 × 10^−3^)
Word	Group x Bundle	1,1800	0.13	0.717	3.47 × 10^−4^ (9.57 × 10^−4^)
	Group	1, 197	1.38	0.241	-0.02 (0.02)
	Bundle	1,1800	16.80	< 0.001	1.79 × 10^−3^ (5.31 × 10^−4^)
Syllable	Group x Bundle	1,1789	13.25	< 0.001	-2.28 × 10^−3^ (7.61 × 10^−4^)
	Group	1,95	3.16	0.079	-0.06 (0.03)
	Bundle	1,1789	9.84	0.002	1.91 × 10^−4^ (4.23 × 10^−4^)

We also investigated the time course of entrainment for the surrogate data (see Table S2; Fig. S3) and compared it to the neural entrainment data. Again, this was done to compare the progression of the true neural entrainment data to the non-entrained oscillatory activity over the exposure period. The results (described in greater detail in the supplementary section) showed that the increase over time that was observed for ITC_word_ and the WLI was not present in the surrogate data, in either adults or children. In addition, direct comparisons indicated that ITC_word_ in the actual entrainment data started at a similar level as in the surrogate data but increased significantly more over time than the surrogate values, for both children and adults. This surrogate comparison thus provides evidence that the increase over time for ITC_word_ and the WLI is a specific index of statistical learning.

## 5 Discussion

In the current study, we probed for possible age-related differences in linguistic SL using both implicit and explicit behavioural tasks, as well as an EEG measure of neural entrainment. To summarise, children showed evidence of SL on all three behavioural measures, as well as on the neural entrainment index. In terms of age-group SL differences, children and adults performed similarly on the 2AFC recognition task and explicit rating tasks, demonstrating comparable levels of explicit knowledge. On the target detection task, while both children and adults showed facilitation for predictable third syllables, adults exhibited relatively *greater* facilitation, as indicated by the RT priming effect. The results from our neural entrainment measure also did not support our original hypothesis that children may have a SL advantage, and provide further nuance in our understanding of the developmental trajectory of SL.

### 5.1 Behavioural evidence of SL in children and adults

While we replicated previous work showing that adults and children perform similarly on explicit measures of linguistic SL (Saffran et al., 1997, McNealy et al., 2010, McNealy et al., 2011, Raviv and Arnon, 2018), a novel contribution of the current study is the use of the target detection task to measure SL in children. Consistent with previous findings in adults, we found that children showed increasingly faster RTs for later, and therefore more predictable, syllable positions, indicating that they used their knowledge of the structure of the speech stream to predict upcoming syllables.

Without correcting for baseline RT differences, both groups also showed similar RT facilitation as a function of syllable position on the target detection task. However, adults showed stronger *relative* facilitation to more predictable third syllables compared to children, as revealed through the RT priming effect, a measure which adjusts for baseline RT differences. Similarly, older children had greater RT priming effects compared to younger children (see Supplementary Materials). Developmental differences in RTs are not completely straightforward to interpret. One possibility is that children and adults accrued similar levels of implicit knowledge, but that older learners were better able to recruit or *express* this knowledge on this task, given that age was also positively associated with objective task performance, including a higher hit rate to targets and fewer false alarms. Alternatively, it may be that older learners acquired stronger implicit knowledge of the statistical structure of the speech stream.

These results are counter to our original hypothesis that children might be better implicit statistical learners and, if anything, point to a slight implicit SL advantage in *older* learners. Although unexpected, our finding that older children and adults express stronger implicit SL effects converges with a previous study of implicit visual sequence learning in 4- to 85-year-olds (Janacsek et al., 2012). After accounting for baseline RTs, adolescents and young adults (11–44 years) showed greater sequence learning relative to younger children (4−10). As we described previously in the Introduction, SL studies using non-linguistic auditory and visual stimuli have also found that older learners outperform younger children on forced-choice recognition measures (Arciuli and Simpson, 2011, Shufaniya and Arnon, 2018).

Interestingly, the pattern of correlations among the tasks also differed between age groups (see Supplementary Material). Children’s performance significantly correlated across tasks, whereas there were no significant inter-task correlations in adults. One possible explanation is that children rely more on implicit memory to complete all tasks. This fits with the two-system model that bottom-up implicit learning develops earlier in life whereas explicit learning, which relies more on top-down attention, develops later in life (Daltrozzo and Conway, 2014). Children in this age range have been shown to perform better on implicit learning tasks than explicit learning tasks (Finn et al., 2015), and thus may rely more on implicit memory when encountering a task that could be supported by either implicit or explicit knowledge. For instance, above-chance recognition judgements on the 2AFC task may be partially or optionally supported by implicit memory (see Voss et al., 2008; Voss and Paller, 2009).

An alternative (and not-mutually exclusive) explanation for these group differences is that domain-general cognitive abilities play a larger role in task performance for children than adults. Children’s ability to successfully execute one experimental task may partially predict their performance on a second task, whereas for adults, task execution is likely to be near ceiling, allowing implicit and explicit memory to dissociate more clearly. This does seem concordant with the previous finding in adults that RT priming on the target detection task does not correlate with recognition task performance (Batterink et al., 2015). However, RT priming has been previously found to correlate with the rating task, in a somewhat larger sample of adults (Batterink and Paller, 2017), suggesting some caution in concluding that two fully dissociable processes are at play.

### 5.2 Neural entrainment evidence of SL in children and adults

As expected, both children and adults showed robust neural entrainment at the word and syllable frequencies (Fig. 4) as well as significant increases in word-level entrainment over time (Fig. 5), reflecting an increased sensitivity to the word structure with more exposure to the language. However, only adults showed a significant increase in the WLI, our ratio measure of word-level relative to syllable-level entrainment; in children, the WLI increase was only marginally significant. Given that word-level entrainment between children and adults showed a highly similar progression, the greater increase in the WLI for adults can be directly linked to differences in syllable entrainment. Syllable-level entrainment showed a strong decrease over time in adults but was relatively stable in children (Fig. 5). These age differences in syllable entrainment over time may reflect developmental changes in other processes that are not directly related to SL, such as attention and auditory habituation. For example, compared to adults, children have been shown to exhibit greatly reduced auditory habituation to trains of simple tones, as measured by MEG responses (Muenssinger et al., 2013). Thus, even though the WLI increase was significantly stronger over time in adults compared to children, it may be premature to conclude that adults were better or more efficient statistical learners, particularly since the progression of word entrainment was highly similar between the two groups. Rather, the influence of broader concepts such as attentional differences between adults and children should be taken into account when comparing neural responses between different ages on an identical task (Muenssinger et al., 2013). Overall, the picture to emerge suggests that adults and children show generally similar SL abilities, even when learning is assessed implicitly and through neural measures. Children entrain to the artificial language somewhat differently than adults, but these entrainment differences do not speak to faster learning or to a SL advantage in children over adults.

Although past studies have found significant correlations between neural entrainment (WLI) and behavioural measures of SL (Buiatti et al., 2009, Batterink and Paller, 2017, Batterink and Paller, 2019, Choi et al., 2020, February 1), we were surprised to see only relatively weak relationships in the present study (see Supplementary Materials). However, this is concordant with several studies reporting significant neural entrainment to word-like units or triplets in the absence of any behavioural evidence of learning (Henin et al., 2021, Farthouat et al., 2017). We also note that an individual’s performance on the various behavioural SL tasks is influenced not only by their true SL abilities, but also by other cognitive processes such as task execution ability, decision making, and memory retrieval (Christiansen, 2019, Kidd et al., 2018), which might obscure correlations between entrainment and behaviour in younger individuals.

### 5.3 Implications for the developmental trajectory of SL

Given our findings, we conclude that SL of sequential speech sounds is robust across the lifespan, and does not decline from childhood to adulthood, unlike many other aspects of language, such as acquisition of phonetic categories (Werker and Tees, 1984, Kuhl et al., 1992) and syntactic rules (e.g., Johnson and Newport, 1991). The conclusion that SL is a mechanism that operates robustly across the lifespan is also supported by the prior SL infant study, in which similar WLI learning dynamics were found over time in infants and adults, suggestive of similar SL trajectories between these two age groups (Choi et al., 2020). While the RT priming effect in the target detection task suggests the possibility of enhanced SL in adults, we did *not* find a clear age-related advantage on our explicit measures of SL—the 2AFC recognition task and the rating task. If adults have a true SL advantage, we would have expected them to outperform children on these measures, given that these more explicit tasks should be biased in favour of adults. However, it also remains possible that adults perform linguistic SL computations slightly more efficiently than children, as has been suggested for non-linguistic SL tasks (Arciuli and Simpson, 2011, Shufaniya and Arnon, 2018).

There are some limitations to our study that should be considered when interpreting our results. The first is regarding differences between groups during the passive exposure phase. Children viewed a silent video, whereas adults in the behavioural group coloured and adults in the EEG group viewed a static image. Because the adult EEG group was a sample of convenience, small differences in the protocol exist. While all these concurrent activities are relatively attentionally non-demanding, it remains possible that the differences between listening conditions may have affected the behavioural and/or neural entrainment results. However, given our general findings showing that both groups had overall similar behavioural and entrainment learning effects, any such effects of concurrent activity during listening seem to be minimal. Future studies should ideally have participants view the same age-appropriate stimuli while passively listening to the auditory stream.

The second limitation relates to the target detection task. Since this was the first time the target detection task was employed in children, syllable presentation was 50 ms slower compared to adults. While this change was intended to make the task easier for children, it may also have inadvertently led to slower responses. This could have impacted the RT priming effect results. However, the overall pattern of results on the target detection task did not differ as a function of age. The take home message here is that both children and adults respond faster to the third syllable compared to the first syllable, demonstrating a facilitation effect of the third syllable. Future research should build on our findings by using identical tasks and stimuli in children and adults in order to confirm our preliminary findings.

Also worth acknowledging is that our youngest participants were eight years old, which limits our understanding of SL in younger children (5–6 years old) who have been previously shown to perform poorly on the explicit 2AFC recognition task (i.e., Raviv and Arnon, 2018). We decided to study 8–12-year-olds on this protocol as (1) existing evidence indicates that this developmental period continues to show advantages for language acquisition relative to adults (e.g., Birdsong, 1999; Johnson and Newport, 1989; Gualtieri and Finn, 2022) and (2) these slightly older children may be more likely to be able to successfully complete the target detection task, which had not been administered before in children. A future direction would be to investigate the SL abilities of younger children using neural entrainment, as well as on the target detection task if feasible, which would provide a more complete understanding of the developmental time course of SL. Further, to make this research more generalisable, future work should test whether these developmental findings hold true in more diverse populations and in non-English speakers.

## 6 Conclusion

To our knowledge, this is the first study to use EEG-based neural entrainment to understand the developmental trajectory and real-time dynamics of SL in children. Our three post-test behavioural measures and time course analysis during learning demonstrated that children and adults rapidly entrained to the words embedded in the language during the six-minute exposure. Crucially, adults and children performed similarly on the behavioural post-test measures and showed similar word-level neural entrainment across time. These results provide evidence against the hypothesis that SL improves over the course of childhood. We conclude that SL is robust across the lifespan, such that children’s advantage for language learning is driven by subcomponents of language other than linguistic SL.

## Declaration of Competing Interest

The authors declare that they have no known competing financial interests or personal relationships that could have appeared to influence the work reported in this paper.

## Data Availability

The data and scripts that support our findings are available on Mendeley Data (https://data.mendeley.com/datasets/3rmh27h4y5/2, doi: 10.17632/3rmh27h4y5.2).

## References

[bib1] Arciuli J. (2017). The multi-component nature of statistical learning. Philos. Trans. R. Soc. B: Biol. Sci..

[bib2] Arciuli J., Simpson I.C. (2011). Statistical learning in typically developing children: the role of age and speed of stimulus presentation. Dev. Sci..

[bib3] Arnon I. (2019). Do current statistical learning capture stable individual differences in children? An investigation of task reliability across modality. Behav. Res. Methods.

[bib4] Aslin R.N. (2017). Statistical learning: a powerful mechanism that operates by mere exposure. Wiley Interdisciplinary Reviews: Cognitive Science.

[bib5] Bates E., Elman J. (1996). Learning rediscovered. (research on language learning by infants). Science.

[bib6] Batterink L.J., Choi D. (2021). Optimizing steady-state responses to index statistical learning: response to Benjamin and colleagues. Cortex.

[bib7] Batterink L.J., Paller K.A. (2017). Online neural monitoring of statistical learning. Cortex.

[bib8] Batterink L.J., Paller K.A. (2019). Statistical learning of speech regularities can occur outside the focus of attention. Cortex.

[bib9] Batterink L.J., Reber P.J., Neville H.J., Paller K.A. (2015). Implicit and explicit contributions to statistical learning. J. Mem. Lang..

[bib10] Benjamin L., Dehaene-Lambertz G., Flo A. (2021). Remarks on the analysis of steady-state responses: spurious artifacts introduced by overlapping epochs. Cortex.

[bib11] Birdsong D. (1999).

[bib12] Buiatti M., Peña M., Dehaene-Lambertz G. (2009). Investigating the neural correlates of continuous speech computation with frequency-tagged neuroelectric responses. Neuroimage.

[bib13] Cary J. (Writer), Symanowski A. (2008). Shaun the Sheep.

[bib14] Chen Y., Jin P., Ding N. (2020). The influence of linguistic information on cortical tracking of words. Neuropsychologia.

[bib15] Choi, D., Batterink, L., Black, A.K., Paller, K., & Werker, J.F.,2020, February 1. Prelingual infants discover statistical word patterns at similar rates as adults: evidence from neural entrainment. 10.31234/osf.io/fuqd2. 32865487

[bib16] Christiansen M.H. (2019). Implicit-statistical learning: a tale of two literatures. Top. Cogn. Sci..

[bib17] Conway C.M. (2020). How does the brain learn environmental structure? Ten core principles for understanding the neurocognitive mechanisms of statistical learning. Neurosci. Biobehav. Rev..

[bib18] Conway C.M., Christiansen M.H. (2005). Modality-constrained statistical learning of tactile, visual, and auditory sequences. J. Exp. Psychol. Learn. Mem. Cogn..

[bib19] Daltrozzo J., Conway C.M. (2014). Neurocognitive mechanisms of statistical-sequential learning: what do event-related potentials tell us*?*. Front. Hum. Neurosci..

[bib20] Delorme A., Makeig S. (2004). EEGLAB: An open source toolbox for analysis of single-trial EEG dynamics. J. Neurosci. Methods.

[bib21] Farthouat J., Franco A., Mary A., Delpouve J., Wens V., Op de Beeck M., De Tiège X., Peigneux P. (2017). Auditory magnetoencephalographic frequency-tagged responses mirror the ongoing segmentation processes underlying statistical learning. Brain Topogr..

[bib22] Finn A.S., Hudson Kam C.L. (2008). The curse of knowledge: first language knowledge impairs adult learners’ use of novel statistics for word segmentation. Cognition.

[bib23] Finn A.S., Kalra P.B., Goetz C., Leonard J.A., Sheridan M.A., Gabrieli J.D.E. (2015). Developmental dissociation between the maturation of procedural memory and declarative memory. J. Exp. Child Psychol..

[bib24] Fiser J., Aslin R.N. (2002). Statistical learning of higher-order temporal structure from visual shape sequences. J. Exp. Psychol.: Learn. Mem. Cogn..

[bib25] Gathercole S.E., Pickering S.J., Ambridge B., Wearing H. (2004). The structure of working memory from 4 to 15 years of age. Developmental Psychology.

[bib26] Gualtieri S., Finn A.S. (2022). The sweet spot: when children’s developing abilities, brains, and knowledge make them better learners than adults. Perspect. Psychol. Sci..

[bib27] Hakuta K., Bialystok E., Wiley E. (2003). Critical evidence: a test of the critical-period hypothesis for second-language acquisition. Psychol. Sci..

[bib28] Hartshorne J., Tenenbaum J., Pinker S. (2018). A critical period for second language acquisition: Evidence from 2/3 million English speakers. Cognition.

[bib29] Henin S., Turk-Browne N.B., Friedman D., Liu A., Dugan P., Flinker A., Doyle W., Devinsky O., Melloni L. (2021). Learning hierarchical sequence representations across human cortex and hippocampus. Sci. Adv..

[bib30] Janacsek K., Fiser J., Nemeth D. (2012). The best time to acquire new skills: age-related differences in implicit sequence learning across the human lifespan. Dev. Sci..

[bib31] Johnson J.S., Newport E.L. (1989). Critical period effects in second language learning: The influence of maturational state on the acquisition of English as a second language. Cogn. Psychol..

[bib32] Johnson J.S., Newport E.L. (1991). Critical period effects on universal properties of language: the status of subjacency in the acquisition of a second language. Cognition.

[bib33] Kidd E., Arciuli J., Christiansen M.H., Isbilen E.S., Revius K., Smithson M. (2020). Measuring children’s auditory statistical learning via serial recall. J. Exp. Child Psychol..

[bib34] Kidd E., Donnelly S., Christiansen M.H. (2018). Individual differences in language acquisition and processing. Trends Cogn. Sci..

[bib35] Kuhl P. (2004). Early language acquisition: cracking the speech code. Nat. Rev. Neurosci..

[bib36] Kuhl P.K., Williams K.A., Lacerda F., Stevens K.N., Lindblom B. (1992). Linguistic experience alters phonetic perception in infants by 6 months of age. Science.

[bib37] Lin C.C.H., Hsiao C.K., Chen W.J. (1999). Development of sustained attention assessed using the continuous performance test among children 6-15 years of age. Journal of Abnormal Child Psychology.

[bib38] Lopez-Calderon J., Luck S.J. (2014). ERPLAB: An open-source toolbox for the analysis of event-related potentials. Front. Hum. Neurosci..

[bib39] McNealy K., Mazziotta J., Dapretto M. (2010). The neural basis of speech parsing in children and adults. Dev. Sci..

[bib40] McNealy K., Mazziotta J., Dapretto M. (2011). Age and experience shape developmental changes in the neural basis of language‐related learning. Dev. Sci..

[bib41] Muenssinger J., Stingl K.T., Matuz T., Binder G., Ehehalt S., Preissl H. (2013). Auditory habituation to simple tones: reduced evidence for habituation in children compared to adults. Front. Hum. Neurosci..

[bib42] Raviv L., Arnon I. (2018). The developmental trajectory of children's auditory and visual statistical learning abilities: modality-based differences in the effect of age. Dev. Sci..

[bib43] Romberg A., Saffran J. (2010). Statistical learning and language acquisition. Wiley Interdiscip. Rev.: Cogn. Sci..

[bib44] Saffran J.R., Newport E.L., Aslin R.N. (1996). Word segmentation: the role of distributional cues. J. Mem. Lang..

[bib45] Saffran J.R., Aslin R.N., Newport E.L. (1996). Statistical learning by 8-month-old infants. N. Ser..

[bib46] Saffran J.R., Newport E.L., Aslin R.N., Tunick R.A., Barrueco S. (1997). Incidental language learning: listening (and learning) out of the corner of your ear. Psychol. Sci..

[bib47] Saffran J.R., Johnson E.K., Aslin R.N., Newport E.L. (1999). Statistical learning of tone sequences by human infants and adults. Cognition.

[bib48] Schlichting M.L., Guarino K.F., Schapiro A.C., Turk-Browne N.B., Preston A.R. (2017). Hippocampal structure predicts statistical learning and associative inference abilities during development. J. Cogn. Neurosci..

[bib49] Seidenberg M.S. (1997). Language acquisition and use: Learning and applying probabilistic constraints. Science.

[bib50] Shufaniya A., Arnon I. (2018). Statistical learning is not age‐invariant during childhood: performance improves with age across modality. Cogn. Sci..

[bib51] Siegelman N., Bogaerts L., Elazar A., Arciuli J., Frost R. (2018). Linguistic entrenchment: prior knowledge impacts statistical learning performance. Cognition.

[bib52] Tallon-Baudry C., Bertrand O., Delpuech C., Pernier J. (1996). Stimulus specificity of phase-locked and non-phase-locked 40 Hz visual responses in human. J. Neurosci..

[bib53] Thiessen E.D., Girard S., Erickson L.C. (2016). Statistical learning and the critical period: how a continuous learning mechanism can give rise to discontinuous learning. Wiley Interdiscip. Rev.: Cogn. Sci..

[bib54] Trecca F., McCauley S., Andersen S., Bleses D., Basbøll H., Højen A., Madsen T., Ribu I., Christiansen M. (2019). Segmentation of highly vocalic speech via statistical learning: initial results from Danish, Norwegian, and English. Lang. Learn..

[bib55] Unsworth N. (2019). Individual differences in long-term memory. Psychol. Bull..

[bib56] Voss J.L., Baym C.L., Paller K.A. (2008). Accurate forced-choice recognition without awareness of memory retrieval. Learn. Mem..

[bib57] Voss J.L., Paller K.A. (2009). An electrophysiological signature of unconscious recognition memory. Nat. Neurosci..

[bib58] Werker J.F., Tees R.C. (1984). Cross-language speech perception: evidence for perceptual reorganization during the first year of life. Infant Behav. Dev..

