# Supplementary material for: No statistical learning advantage in children over adults: Evidence from behaviour and neural entrainment
Source: Dev Cogn Neurosci. 2022 Sep 21;57:101154. doi: 10.1016/j.dcn.2022.101154 (PMC9507983; doi:10.1016/j.dcn.2022.101154)
Supplement: Supplementary file 1 — Supplementary material. [file mmc1.docx]

**Supplementary Material**

**Behavioural Results**

**Remember/know Task**

After each 2AFC, following Batterink et al. (2015), the participants were asked to provide a remember/familiar/guess response in which “remember” indicates they specifically remembered hearing the word, “familiar” indicates that they did not specifically remember hearing the word, but that it sounded familiar, and “guess” indicates that they had no confidence in their response. A repeated measures ANOVA was conducted to determine whether familiarity judgement differed according to accuracy and whether there were significant differences across groups.

Accuracy did not significantly differ as a function of familiarity judgement for the children and the adults (Remember/know effect: Children: *F*(2, 82) = 2.194,*p* = .118, ***η****_p_^2^*= .051; linear contrast: *F*(1, 41) = 2.382, *p* = .130, ***η****_p_^2^*= .055; Adults: *F*(1.512, 36.728) = 1.592, *p* = .153, ***η****_p_^2^* = .075; linear contrast: *F*(1, 24) = 1.670, *p* = .209, ***η****_p_^2^* = .065). However, statistical power was limited as 13 children did not choose “guess” and 15 adults did not choose “remember” or “guess”. Remember, familiar, and guess categories were significantly above chance for the children (remember: *t*(54) = 6.614, *p* < .001, Cohen’s d = 0.892; familiar: *t*(53) = 4.552, *p* < .001, Cohen’s d = .619; guess: *t*(41) = 2.577, *p* = .014, Cohen’s d = .398). In contrast, for the adults, the “remember” and “familiar” categories were significantly above chance; whereas “guess” was not (*t*(37) = 4.943, *p* < .001, Cohen’s d = .802; *t*(39) = 2.319, *p* = .026, Cohen’s d = .367; *t*(26) = .907, *p* = .372, Cohen’s d = .175, respectively). See Figure 1.

Familiarity judgements did not differ according to accuracy. We expected that the remember response would have higher accuracy than the familiar and guess responses. These findings are divergent from Batterink and colleagues’ (2015) findings of a significant effect in adults. One explanation for this is that we had fewer remember/know trials than Batterink et al. (2015). Their study had 36 trials, whereas our study had 16 trials. This could have affected the task’s sensitivity to detect a significant meta-memory effect.

**Figure S1**

Boxplots of percent correct on the 2AFC task for the remember, familiar, guess categories on the remember/know for adults and children. The middle line is the 50% quantile. The lower hinge is the 25% quantile and the upper hinge is the 75% quantile. The lower whisker is the smallest observation greater than or equal to the lower hinge - 1.5 * Interquartile Range (IQR). The upper whisker is the largest observation less than or equal to the upper hinge + 1.5 * IQR.


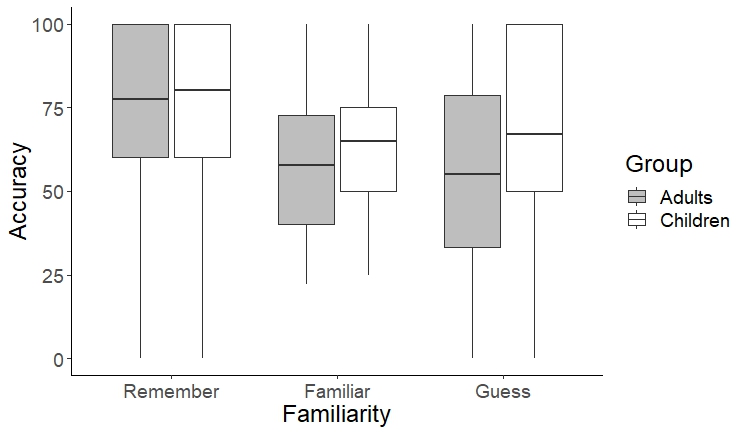


**Rating Task**

The rating task effect was significant for adult sample A, indicating that they rated words greater than partwords, followed by nonwords (Word Category effect: *F*(2, 38) = 15.85, *p* < .001, ***η****_p_^2^* = .455; linear contrast: *F*(1,19) = 24.09, *p* < .001, ***η****_p_^2^* = .559). This effect did not significantly differ between adult sample A and children (Word Category x Group effect: *F*(2, 146) = 1.76, *p* = .175, ***η****_p_^2^* = .024; linear contrast: *F*(1,73) = 1.72, *p* = .194, ***η****_p_^2^* = .023).

**Target Detection Task**

Adult sample A responded significantly faster to the last syllable compared to the second and the first syllable (Syllable Position Effect: *F*(2, 38) = 66.69, p < .001, ***η****_p_^2^* = .777; linear contrast: *F*(1,19) = 101.33, *p* < .001, ***η****_p_^2^* = .842). No significant difference in this effect was found between adult sample A and the children (Syllable Position x Group effect: *F*(2, 146) = .49, *p* = .615, ***η****_p_^2^* = .007; linear contrast: *F*(1,73) = .233, *p* = .631, ***η****_p_^2^* = .003).

**EEG Results**

**Overall Neural Entrainment Effects across Exposure**

**Figure S2**

Overall entrainment (green) vs. surrogate data (purple) for adults (A) and children (B). No clear peaks are found at the syllable and word frequencies for the surrogate data. Results from statistical testing between entrainment and surrogate data are reported in the main manuscript. No significant differences in surrogate values were found between children and adults (Word: t(77) = .59, p = .554, Cohen’s d = .145; Syllable: t(77) = 1.87, p = .065, Cohen’s d = .458).

**
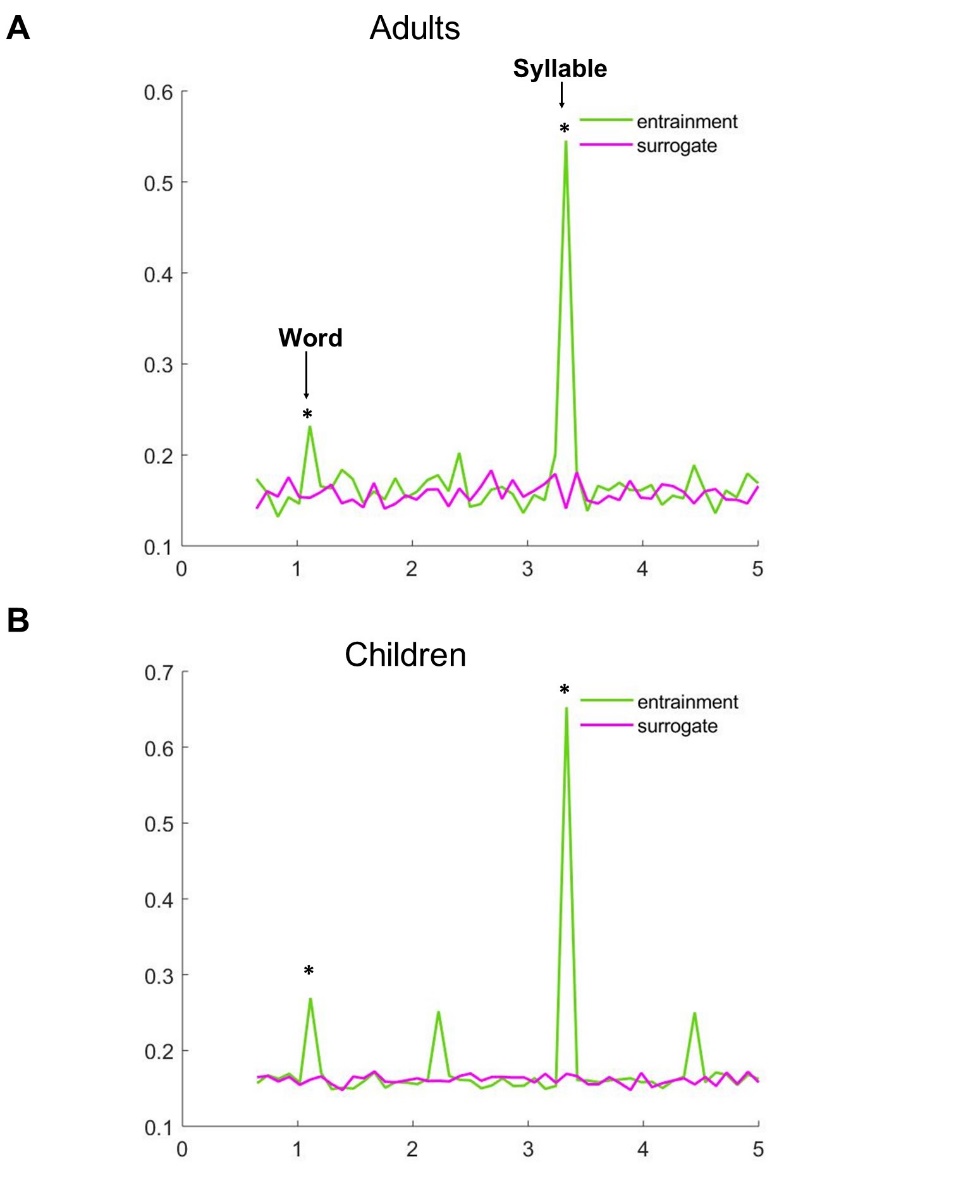
**

**Table S1**

*Mean and standard deviations for the overall entrainment effects. Actual and surrogate ITC values are reported for children and adults.*

| Group | Word | | Syllable | |
| --- | --- | --- | --- | --- |
|  | Actual | Surrogate | Actual | Surrogate |
| Children | .27 (.09) | .16 (.06) | .65 (.16) | .17 (.07) |
| Adults | .23 (.09) | .15 (.06) | .55 (.15) | .14 (.05) |

**Time Course of Neural Entrainment**

To test whether the changes in neural entrainment over time are a specific index of statistical learning, the same time course analysis that was applied to the actual entrainment data was also conducted on the surrogate data. As described in 2.3.1 of the main manuscript, a surrogate dataset was created for each participant by jittering each word onset relative to the actual word onset and extracting epochs to these randomly jittered word onsets. Within each age group, the same mixed effect model (described in 2.3.2, with bundle as fixed effect and participant as random intercept) that was applied to the original entrainment data was than applied to the surrogate data, for ITC_word_, ITC_syllable_ and the WLI (i.e., six models total). Table S2 demonstrates the time course of these surrogate ITC values. Children showed no significant changes in ITC_word_ over time, and a significant increase in ITC_syllable_ over time, and a significant decrease in WLI. Adults showed a significant decrease in ITC_Word_ over time and no change in ITC_Syllable_ or WLI over time. Thus, the increase in ITC_word_ (and the WLI) that both adults and children showed in the actual entrainment data was not observed in the shuffled data.

Figure S3 directly compares the time course of the actual entrainment data and to the surrogate data. To directly compare the time course of neural entrainment between the entrainment and surrogate data, within each age group, three separate models were tested that included ITC_word_ (or ITC_Syllable_ or WLI) as the dependent variable, bundle, condition (actual, surrogate), and the interaction between bundle and condition as fixed effects, and participant as a random intercept. The results of this model are shown in Table S3. There were significant differences between the actual and surrogate data for the time course of ITC_word_ and the WLI; however, the time course of ITC_syllable_ was not significantly different in either group. The results are further interpreted in the Main Manuscript.

**Table S2**

*Time course of surrogate data in children and adults. The table reports the key linear mixed-effects model results for the ITC_word_ and ITC_syllable_ reported by group (children and adults). Parameter estimate indicates the increase or decrease as a function of bundle.*

| Group | ITC | *df* | *F* | *p* | Parameter estimate (SE) |
| --- | --- | --- | --- | --- | --- |
| Children | Word | 1, 1359 | .60 | .439 | 3.24 x 10^-4^ (4.18 x 10^-4^) |
|  | Syllable | 1, 1372 | 12.57 | <.001 | 1.63 x 10^-3^ (4.58 x 10^-4^) |
|  | WLI | 1, 1370 | 4.50 | .034 | -4.15 x 10^-3^ (1.96 x 10^-3^) |
| Adults | Word | 1, 577 | 3.87 | .050 | -1.52 x 10^-3^ (7.72 x 10^-4^) |
|  | Syllable | 1, 575 | 1.57 | .210 | 9.40 x 10^-4^ (7.50 x 10^-4^) |
|  | WLI | 1, 575 | .61 | .434 | 2.57 x 10^-3^ (3.28 x 10^-3^) |

**Figure S3**

Time course of actual vs. surrogate data for (A) children’s word entrainment, (B) children’s syllable entrainment, (C) children’s WLI, (D) adults’ word entrainment, (E) adults’ syllable entrainment, and (F) adults’ WLI.


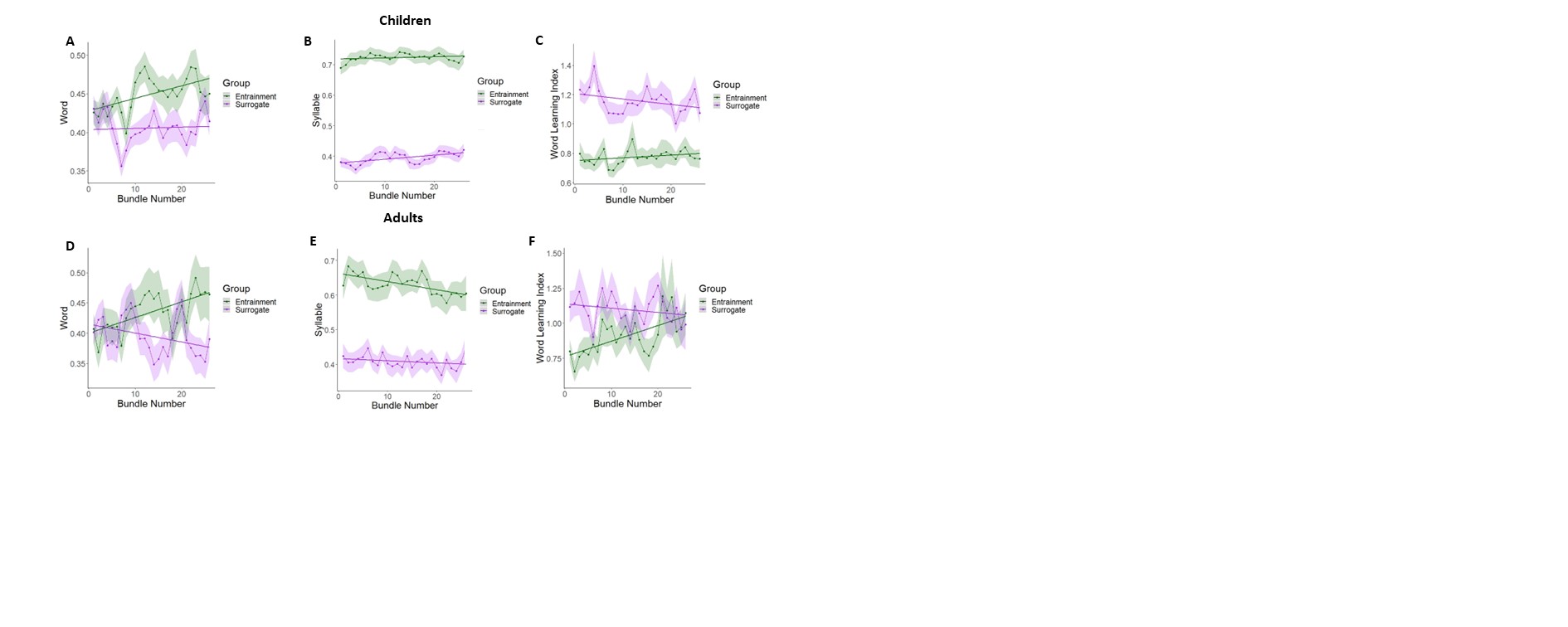


**Table S3**

*Results of the model comparing time course of actual vs. surrogate data in children and adults. The table reports the key linear mixed-effects model results for ITC_word_, ITC_syllable_ and WLI, within each group (children and adults). Parameter estimates indicate the interaction between condition (actual, surrogate) and bundle, reflecting whether the time course of ITC significantly differed between actual entrainment and surrogate data. Positive values indicate an increase in the time course of the actual vs. the surrogate data.*

| Group | ITC | *df* | *F* | *p* | Parameter estimate (SE) |
| --- | --- | --- | --- | --- | --- |
| Children | Word | 1, 2716 | 5.22 | .022 | 1.44 x 10^-3^ (6.29 x 10^-4^) |
|  | Syllable | 1,2715 | 3.15 | .076 | -1.11 x 10^-3^ (6.28 x 10^-4^) |
|  | WLI | 1, 2714 | 7.54 | .006 | 5.95 x 10^-3^ (2.17 x 10^-3^) |
| Adults | Word | 1,1153 | 12.06 | < .001 | 4.06 x 10^-3^ (1.17 x 10^-3^) |
|  | Syllable | 1,1154 | 1.77 | .184 | -1.56 x 10^-3^ (1.17 x10^-3^) |
|  | WLI | 1, 1154 | 7.94 | .005 | .01 (3.96 x 10^-3^) |

**The Impact of Age on the Time Course of Entrainment within Children**

To test the impact of age on neural entrainment within the child sample, three separate mixed effect models were conducted, with ITC_word_, ITC_Syllable_ or WLI as the dependent variable, bundle, age, and the interaction between bundle and age as fixed effects, and participant as a random intercept. The results are reported in Table S4. ITC_word_ was found to increase significantly more over time in older children. However, ITC_syllable_ and the WLI did not change significantly according to age.

**Table S4**

*Linear mixed-effects model for age-related differences within children (in months) for WLI, ITC_word_, ITC_syllable_. The age x bundle interaction is reported. Positive parameter estimates for the interaction indicate greater entrainment for older children. Intercept age in the model is theoretically 0 months.*

| ITC | df | F | p | Parameter estimate (SE) | Interpretation |
| --- | --- | --- | --- | --- | --- |
| WLI | 1,1244 | 2.44 | .119 | 1.70 x 10^-4^ (1.09 x 10^-4^) | No difference across age |
| Word | 1,1252 | 7.01 | .008 | 9.30 x 10^-5^ (3.51 x 10^-5^) | Stronger increase over time in older ages |
| Syllable | 1,1239 | 2.51 | .113 | 4.09 x 10^-5^ (2.58 x 10^-5^) | No difference across age |

**Age and Inter-Task Correlations Within Children**

Within our child sample, age did not correlate significantly with our more explicit behavioural measures [rating score: (*r*(53) = .26, *p* = .057), recognition accuracy (*r*(54) = .08, *p* = .550)]. We also investigated the word category effect for the rating task by adding age as a covariate in the original ANOVA. Performance did not differ according to age (*F*(1, 53) = 3.92, *p* = .053, ***η****_p_^2^* = .069), though we note that this effect is in the marginal range, suggesting that older children may have performed somewhat better on this task. However, age significantly correlated with RT priming (*r*(53) = .28, *p* = .037), with older children having a greater RT priming score. This result is consistent with the larger RT priming effect in adults compared to children as a group. The syllable position effect was also investigated by adding age as a covariate to the original ANOVA. Similar to the RT priming score, this effect also differed as a function of children’s age (*F*(1, 53) = 16.75, *p* < .001, ***η****_p_^2^* = .240), indicating that older children responded faster to final syllables in comparison to younger children. Age also strongly correlated with hit rate on the target detection task (*r*(54) = .43, *p* = .001), as well as overall speed (*r*(53) = -.49, *p* < .001), with older children having a greater hit rate and faster RT. Performance across all three behavioural measures were correlated for children (Table S3) but not in adults (Table S4; see also Figure S3).

**Figure S4**

Inter-task behavioural correlations in children and adults. A, D) Correlation between the 2AFC accuracy scores and RT priming. B, E) Correlations between the 2AFC accuracy score and the rating score. C, F) Correlations between RT priming and the rating score. Note: there are 40 participants included in F and 20 included in D and E because only half of the sample were given the 2AFC task. * p < .05, **p < .01.

*
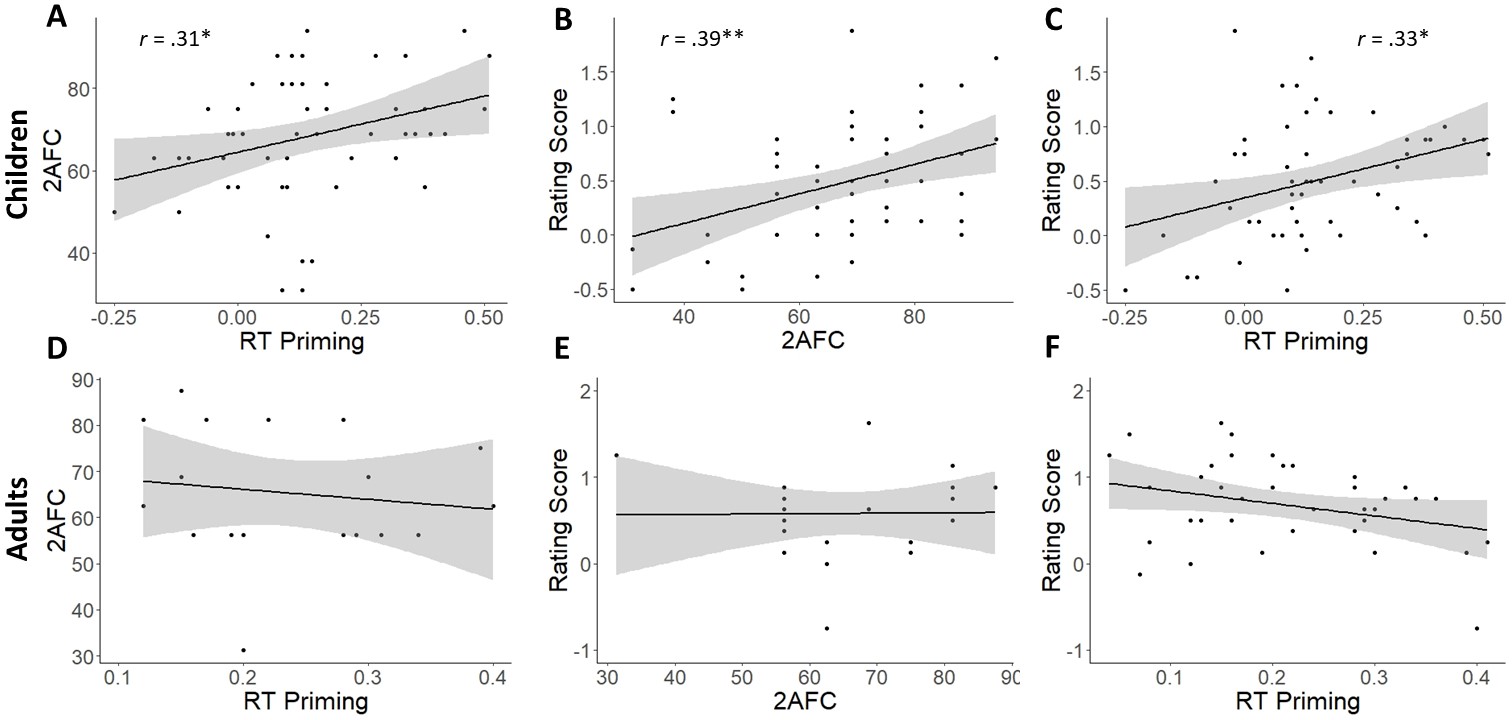
*

**Behaviour-Neural Entrainment Correlations**

Counter to our hypotheses and previous findings, the WLI did not significantly predict subsequent performance on any task, in either the children or the adults (see Table S3 and Table S4). However, the WLI showed a marginal positive correlation with performance on the rating task in children.

**Table S5**

*Correlations between the neural and behavioural measures for the children. Significant correlations are bolded.*

|  | WLI  *(n = 55)* | Rating task  *(n = 55)* | 2AFC  *(n = 55)* |
| --- | --- | --- | --- |
| Rating task | .26, *p* = .061*†* | -- |  |
| 2AFC | .09, *p* = .511 | **.39, *p* = .004**** | -- |
| RT priming | .02, *p* = .901 | **.33, *p* = .016*** (n = 54) | **.31, *p =* .021*** |

*Notes. †* < 0.1, **p* < .05, ***p* < .01, *p* < .001***

**Table S6**

*Correlations between the neural and behavioural measures in adults. Significant correlations are bolded.*

|  | WLI  *(n = 20)* | Rating task  *(n = 40)* | 2AFC  *(n = 20)* |
| --- | --- | --- | --- |
| Rating task | -.08, *p* = .709 | -- | .01, *p* = .960 |
| RT priming | .28, *p* = .191 | -.30, *p* = .059*†* | -.20, *p* = .394 |

*Note. †* < 0.1, **p* < .05, ***p* < .01, *p* < .001***
